# Creosote Bush (*Larrea tridentata*) Improves Insulin Sensitivity and Reduces Plasma and Hepatic Lipids in Hamsters Fed a High Fat and Cholesterol Diet

**DOI:** 10.3389/fphar.2016.00194

**Published:** 2016-06-28

**Authors:** Georgina Del Vecchyo-Tenorio, Maricela Rodríguez-Cruz, Adolfo Andrade-Cetto, René Cárdenas-Vázquez

**Affiliations:** ^1^Laboratorio de Biología Animal Experimental, Departamento de Biología Celular, Facultad de Ciencias, Universidad Nacional Autónoma de MéxicoMexico City, Mexico; ^2^Laboratorio de Nutrición Molecular, Unidad de Investigación Médica en Nutrición, Hospital de Pediatría, Centro Médico Nacional Siglo XXI, Instituto Mexicano del Seguro SocialMexico City, Mexico; ^3^Laboratorio de Etnofarmacología, Departamento de Biología Celular, Facultad de Ciencias, Universidad Nacional Autónoma de MéxicoMexico City, Mexico

**Keywords:** *Larrea tridentata*, cholesterol, hamster, high fat diet, insulin tolerance

## Abstract

Creosote bush, *Larrea tridentata* (Sesse y Moc. Ex DC, Zygophyllaceae) is a shrub found in the deserts of Northern Mexico and Southwestern United States. In traditional medicine, it is used to treat a variety of illnesses including type 2 diabetes. The present study aims to investigate the effects of creosote bush ethanolic extract on plasma and liver parameters associated with the metabolic syndrome in hamsters fed a high fat and cholesterol diet (HFD), comparing them with those induced by ezetimibe (EZ). Seven groups of six hamsters each were formed. Six groups were fed HFD for 2 weeks. The following 2 weeks, the HFD groups received: (1) only HFD, (2) HFD + 3 mg% EZ, (3) HFD + 0.2% creosote bush ethanolic extract, (4) only standard diet (Std Diet), (5) Std Diet + 3 mg% EZ, (6) Std Diet + 0.2% creosote bush ethanolic extract. The beneficial effects of creosote bush ethanolic extract in the HFD hamster model were a reduction of insulin resistance, associated with lower serum insulin and leptin, lower hepatic lipid peroxidation and higher liver antioxidant capacity. Plasma and liver lipids tended or were reduced to values closer to those of animals fed standard diet. A similar effect on lipids was induced by EZ, although with even lower hepatic cholesterol and total lipids concentrations. In general, the change from HFD to standard diet plus ethanolic extract induced the same but deeper changes, including a reduction in plasma glucose and an increase in the percentage of HDL cholesterol. Unlike creosote bush extract, EZ increased food consumption and neutral fecal steroids, with no significant effect on body weight, epididymal fat pads, liver peroxidation or antioxidant capacity. Also EZ did not modify serum insulin and leptin. However, insulin sensitivity improved to values similar to those induced by the extract. This suggests that the mechanism of action of creosote bush ethanolic extract is different to inhibition of cholesterol absorption or increase excretion. The ethanolic extract of *L. tridentata* could be useful in the treatment of the metabolic syndrome.

## Introduction

Creosote bush, *Larrea tridentata* (Sesse y Moc. Ex DC, Zygophyllaceae) is a shrub found in the deserts of Northern Mexico and Southwestern United States. The resin that covers the leaves contains flavonoid aglycones, as well as several lignans, notably including the antioxidant NDGA (Konno et al., 1990). Other lignans present are the linear ones, guaiaretic acid and meso-dihydroguaiaretic acid, and the cyclolignans, norisoguaiacin and it’s 3 methyl derivative (Gnabre et al., 2015). *L. tridentata* contains about 0.1% of dry weight as volatile oils, mainly monoterpenoids and aromatic sesquiterpenoids (Mabry and Bohnstedt, 1981; Xue et al., 1988). The ethanol extraction of leaves and twigs of *L. tridentata*, at room temperature, yields 10% resin in a dry weight basis, containing 26% NDGA (Belmares et al., 1981).

Creosote bush is used to treat a variety of illnesses including type 2 diabetes. Oral decoctions and extracts of leaves and twigs have been used by the Pima Indians in the United States and in Mexico for the treatment of diabetes (Winkelman, 1989). Additionally, it has been reported that NDGA reduces plasma glucose and TGs in rats treated with streptozotocin (Luo et al., 1998; Reed et al., 1999); it also reduces TG secretion and liver TG content in rats with fructose-induced hypertriglyceridemia (Scribner et al., 2000) and inhibits dipeptidyl peptidase 4 (Roskar et al., 2016). Many of the pharmacological activities of *L. tridentata* have been ascribed to its lignans, although many other components are present in the extract that could be synergizing or potentiating its activities. Thorough reviews dealing with the medicinal uses and phytochemistry of creosote bush and NDGA are available (Arteaga et al., 2005; Gnabre et al., 2015).

A risk factor for developing type 2 diabetes and cardiovascular disease, is the MS. Central obesity and insulin resistance are acknowledged as important causative factors. According to the IDF (Alberti et al., 2006), MS includes central obesity and two of the following factors: decreased HDL cholesterol, elevated TGs, high blood pressure and raised fasting glucose. Hyperleptinemia, due to leptin resistance, may also be an important etiological component of the MS (Wang et al., 2010). Persons with MS are predispose to develop fatty liver and its complications (Wang et al., 2010).

Since MS is a group of metabolic abnormalities that recently have been characterized, it is difficult to find in the Mexican traditional medicine plants specifically used for its treatment. However, creosote bush is used to control hyperglycemia and hyperlipidemia, two key factors of the MS (Winkelman, 1989).

The aim of the present work was to study the effects of creosote bush ethanolic extract on the Syrian golden hamster fed a high fat and cholesterol diet that expresses some components of the MS, in order to determine its possible use in the treatment of this alteration. Since change of life style, mainly diet and exercise, is recommended for treatment of MS in humans, the effect of changing high fat diet to maintenance diet with or without creosote bush ethanolic extract was tested. It was expected that the change of diet plus extract could induce higher improvement of the altered parameters.

## Materials and Methods

### Plant Extract

The plants of *L. tridentata* used to prepare the ethanolic extract were collected at San Luis Potosi State, Mexico. Voucher specimens were deposited at the National Herbarium, Institute of Biology, UNAM (MEXU; no. 534807) and at the Herbarium of Medicinal Plants, S. XXI Medical Center (IMSS; nos 11 319–11 321).

The ethanolic extract was prepared from 100 g of coarsely fragmented leaves and twigs, extracted in 1 L absolute ethanol, mixed for 30 min at room temperature, filtered with paper and reduced to approximately 40 mL in a rotary evaporator under reduced pressure and then lyophilized. The ratio of the herbal drug to the herbal drug preparation (DER native) was 10:1.

### NDGA Content in the Ethanolic Extract

**FIGURE 1:**
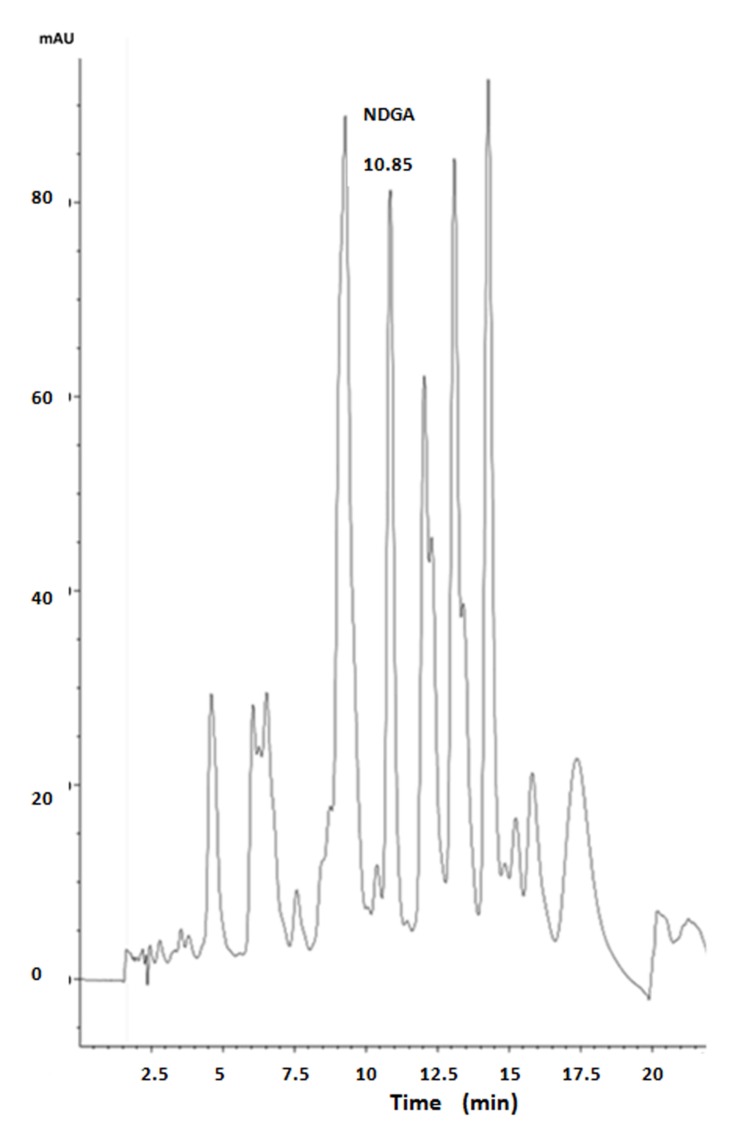
**HPLC chromatogram of creosote bush extract**.

For the HPLC analysis, an Agilent 1260 Infinity system with diode array detector and a Macherey–Nagel 100-5 C18 (250 mm × 4.6 mm) column were employed, with H_3_PO_4_ 0.04 M and acetonitrile (ACN) mixtures as mobile phase, at a flow rate of 1.5 ml/min and 20 μl sample volume injection. Initially a relation of H_3_PO_4_/ACN 70:30 (v/v) was started, with ACN concentration being increased up to 50% by minute 15. Then ACN was increased again, so that a relation of H_3_PO_4_/ACN 30:70 (v/v) was reached by minute 19. Finally, mobile phase was returned to an ACN proportion of 30% by minute 21. NDGA (Sigma–Aldrich) and creosote bush ethanolic extract were dissolved in methanol. An NDGA standard curve was made in the range 20–80 μg/ml, following absorbance at 280 nm. As shown in the chromatogram (**Figure 1**), the creosote bush extract contains NDGA (8.9%), together with several other peaks, which could be *O*-methylated derivatives that have been reported in creosote bush (Gnabre et al., 2015).

### Animals and Diets

Thirty-six male Syrian hamsters, 5-week-old, weighing 72 g were obtained from the stock colony kept at the animal house from the Faculty of Sciences, UNAM, Mexico, were fed *ad libitum* with a HFD containing standard rodent chow (Std diet: Purina Nutricubes; 3.39 kcal/g) 70%, casein 6.9%, cellulose 1.5%, lard 20%, methionine 0.12%, choline 0.042%, AIN-93M Mineral Mix 1.05%, AIN-93Vitamin Mix 0.3% and cholesterol 0.1%. The energy content was 4.46 kcal/g diet. The control group (*n* = 6) was fed with the standard rodent chow. After 2 weeks, the hamsters on HFD were randomly divided in six groups of six animals each with average similar body weight and fed the following diets and water *ad libitum*: (1) HFD; (2) HFD with added ezetimibe (EZ; Zient, Schering-Plough) at 3 mg% as a positive control; (3) HFD added with creosote bush ethanolic extract at 0.2%; (4) standard rodent chow (Std diet); (5) Std diet added with creosote bush ethanolic extract at 0.2%, and (6) Std diet with 3 mg% EZ. The level of extract included in the HFD diet was based upon the result of a preliminary experiment indicating a higher and more consistent effect on plasmatic lipids than with a lower one (0.1%). A higher level (0.25%) was equally effective. EZ was chosen as a positive control at 3 mg% of diet (∼2 mg/kg b.w.), since it had been reported that this dose is effective in normalizing the combined dyslipidemia induced by high fat diet in male hamsters (van Heek et al., 2001). Each group was housed in a stainless steel cage with sawdust bedding, at 22 ± 2°C, under a 14:10 h light–dark cycle. Food consumption was measured every other day.

The animals were handled according to the Health Guide for the Care and Use of Laboratory Animals (Office of animal care and use of the National Institute of Health, 1996). All methods used in this study were approved by the Internal Council of the Facultad de Ciencias of the Universidad Nacional Autónoma de México.

### Blood and Tissues Sample Collection

At the end of the fourth week the animals were fasted for 4 h, anesthetized with pentobarbital (50 mg/kg b.w. i.p.) and blood samples were taken from the retro-orbital sinus with heparinized and non-heparinized capillary tubes. An insulin tolerance test was then performed as shown below. After the insulin tolerance test, the animals were euthanized with a pentobarbital overdose. The liver and epididymal fat depots were dissected, weighed and frozen at –40°C. Plasma and serum was obtained by centrifugation on a hematocrit centrifuge and kept at –40°C.

### Plasma and Serum Analysis

Plasma glucose, TGs, total cholesterol, and HDL cholesterol were determined with enzymatic-colorimetric assay kits (SpinReact, Girona, Spain). Insulin and leptin serum concentrations were determined by ELISA Kits (Millipore Rat/Mouse Insulin, Rat Leptin).

### Insulin Tolerance Test

Under anesthesia the insulin tolerance test (ITT) was performed with an insulin injection (i.p.) at a dose of 0.2 IU/100 g body weight, blood from the retro-orbital sinus was sampled at 0, 5, 10, and 15 min after insulin injection. Plasma glucose was determined with an enzymatic-colorimetric assay kit, as above.

### Liver Parameters

A liver sample was homogenized in a Dunce apparatus with methanol:chloroform (1:1) and centrifuged at 1000 × *g* for 5 min at 4°C. Supernatant samples were evaporated under vacuum and total lipids measured by the phospho-vanillin method (Inouye and Lotufo, 2006), TGs and cholesterol by enzymatic colorimetric assays (SpinReact, Girona, Spain).

A 10% liver homogenate in saline was prepared and centrifuged at 1000 × *g* for 10 min at 4°C. Liver peroxidation was determined by Thiobarbituric Acid Reactive Substances method (TBARs; Ohkawa et al., 1979) and antioxidant capacity by DPPH assay (Hsu et al., 2007; Sharma and Bhat, 2009) in the homogenate supernatant.

### Neutral Fecal Sterols

The neutral fecal sterols were extracted with chloroform:methanol:saline (2:1:0.75) from samples of 72 h feces collected at the end of the second week of treatments. Feces were dried, powdered and homogenized. Sterols were determined in the dried organic phase by the Lieberman–Burchard reaction (Richterich and Colombo, 1981), with cholesterol as standard.

### Statistical Analyses

Statistical analyses and graphics were performed with GraphPad Prism version 6.00 for Windows (GraphPad Software, La Jolla, CA, USA). All data are reported as mean ± SD for the specified number of samples. Differences between mean values were tested for statistical significance (*P* < 0.05) by 1-way ANOVA. Tukey HSD was used as *post hoc* test. Pearson correlation was performed between body weight and epididymal fat depots.

## Results

### Body Weight, Epididymal Fat Depots, and Food Consumption

After the first 2 weeks on HFD, six groups of similar body weight were formed. Although these groups had higher body weight, it was not significantly different from that of the Std diet fed group (**Table 1**). After 4 weeks, significant (*P* < 0.05) differences in body weight among groups were found. Animals on HFD were significantly heavier than those on Std diet. Groups with EZ, creosote bush ethanolic extract and those changed from HFD to Std diet with or without EZ were not significantly different from the heaviest HDF group nor from the lighter one that receiving only Std diet for 4 weeks. The group changed from HFD to Std diet with extract exhibited a significantly (*P* < 0.05) lower body weight than that of the HFD group and very similar to the one fed Std diet for 4 weeks. Epididymal fat depots followed the same pattern, except that the lighter group was the one that changed to Std diet added with extract. A significant correlation was found between body weight and epididymal fat weight (*r* = 0.8, *n* = 42, *P* < 0.001).

**Table 1:** Body weight and food consumption in hamsters^∗^.

Groups and diets	Body weight (g) at 2 weeks	Body weight (g) at 4 weeks	Epididymal fat pads (g)	Food consumption (g/animal/day)^1^
Std. diet (4 weeks)	97,2 ± 7,2ˆa	111,3 ± 9,7ˆa	1,43 ± 0,29ˆa,c	7,9 ± 1.0ˆa,b
HFD (4 weeks)	109,8 ± 10,5ˆa	133,5 ± 10,5ˆb	1,90 ± 0,28ˆb	8,5 ± 0.3ˆa,b,d
HFD + 3mg% ezetimibe	109,2 ± 7,4ˆa	126,5 ± 11,0ˆa,b	1,88 ± 0,25ˆa,b	9,2 ± 0.5ˆa,d
HFD + 0.2% ethanolic extract	109,0 ± 8,9ˆa	119,7 ± 10,4ˆa,b	1,43 ± 0,18ˆa,c	6,8 ± 0.8ˆb
HFD→Std. diet	108,8 ± 6,0ˆa	120,2 ± 5,5ˆa,b	1,50 ± 0,18ˆa,b,c	7,9 ± 0.4ˆa,b
HDF→Std. diet + 3 mg% ezetimibe	108,8 ± 10,8ˆa	125,0 ± 14,0ˆa,b	1,67 ± 0,35ˆa,b	14,1 ± 2.2ˆc
HFD→Std. diet + 0.2% ethanolic extract	108,8 ± 11,3ˆa	112,2 ± 10,5ˆa	1,06 ± 0,22ˆc	9,8 ± 1,6ˆd

*^∗^Mean ± SD, n = 6 per group. Different letter in the same column indicate a statistical difference (P < 0.05; ANOVA and Tukey). ^1^Food consumption on last 2 weeks, n = 8 per group.*

In relation to food consumption, there were significant differences among groups. The lowest food consumption among the groups with HFD for the last 2 weeks was the one with HFD and *L. tridentata* extract, but consumption was not significantly different from the groups fed either HFD or Std diet for 4 weeks. The change from HFD to Std diet did not differ in food consumption from those with Std for 4 weeks nor the one with HFD. The change to Std diet plus extract induced a significantly higher consumption than the change to Std diet without additions. The group that changed from HFD to Std diet and EZ exhibited the significantly highest food consumption of all groups (*P* < 0.05).

### Plasma Parameters

**FIGURE 2:**
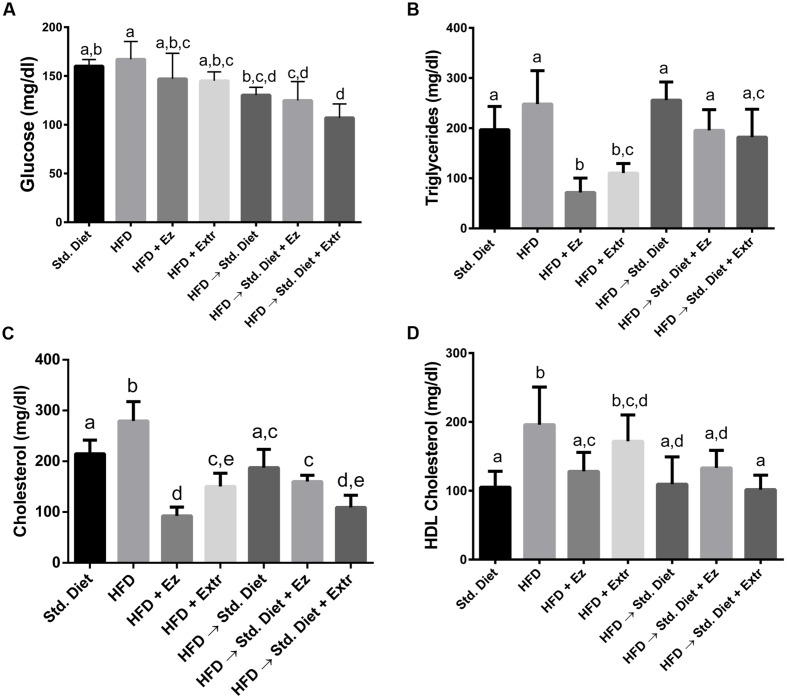
**Plasma (A) glucose, (B) triglycerides, (C) cholesterol, and (D) HDL cholesterol in hamsters fed HFD for 2 weeks and Std diet or HFD diet with or without creosote bush extract or ezetimibe (EZ) for another 2 weeks**. Mean ± SD, *n* = 6 per group. Different letter between columns means significant difference (*P* < 0.05).

At the end of the treatment period, there were significant differences in plasma parameters among groups (**Figure 2**). Glucose was not significantly different among groups fed only Std diet or HFD with or without any addition. Also, no differences were found among the three groups changed to Std diet. However, addition of EZ or creosote bush extract induced lower glucose concentrations in the groups changed to Std diet when compared with Std diet and HFD controls (*P* < 0.05).

In TGs, no significant difference was found between Std diet and HFD control groups. The addition of EZ and extract to HFD lower this parameter (*P* < 0.05). The change to Std diet with or without additions did not significantly altered TGs. Total cholesterol was higher in HFD control group (*P* < 0.05). Both EZ and extract additions to HFD induced lower concentration than those of the HFD and Std diet controls. The change to Std diet return cholesterol level to Std diet control concentrations. Creosote bush extract in the Std diet significantly reduced cholesterol, whereas EZ addition did not when were compared with the group on HFD changed to Std diet.

In relation to HDL cholesterol, groups with HFD, except the one with EZ, exhibited higher values than the Std diet control group. The change to Std diet reduced HDL cholesterol to values significantly (*P* < 0.05) different from the HFD control group. Addition of EZ or extract to the Std diet had no effect on HDL cholesterol, when compared with the group that change to Std diet.

### Serum Insulin and Leptin

**FIGURE 3:**
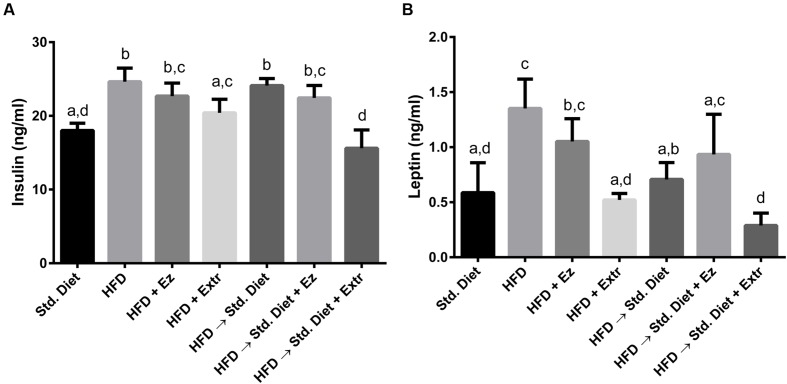
**Serum insulin (A) and leptin (B) concentrations in hamsters fed HFD for 2 weeks and Std diet or HFD diet with or without creosote bush extract or EZ for another 2 weeks**. Mean ± SD, *n* = 6 per group. Different letter between columns means significant difference (*P* < 0.05).

As shown in **Figure 3**, there were increments in both insulin and leptin serum concentrations in groups fed with HFD and HFD plus EZ. Added to HFD, the ethanolic extract reduced leptin and insulin to values significantly (*P* < 0.05) different from those obtained with HFD.

The change from HFD to Std diet reduced leptin but not insulin concentration. Addition of EZ did not induce a significant reduction of any of the two hormones from the concentrations found in the HFD group. The change to Std diet added with ethanolic extract induced the lowest levels of leptin and insulin of all groups, although they were not significantly different from those found in the group with Std diet for 4 weeks.

### Insulin Tolerance Test

There were significant differences in the sensitivity to an insulin injection, expressed as the percentage reduction of the initial glucose concentration per minute (**Table 2**). The HFD group showed the lowest sensitivity to insulin. The additions of ethanolic extract of creosote bush or EZ to the HFD induced significant (*P* < 0.05) increases in insulin sensitivity, which were not different from the group fed Std diet. The change from HFD to Std diet similarly increased the sensitivity to insulin to an intermediate value that was not different from that of the control group receiving Std diet for 4 weeks. The change from HFD to Std diet added with EZ further increased sensitivity to a value not significantly different from either the control group fed Std diet for 4 weeks or the group changed to Std diet. The highest sensitivity to insulin was induced by the change from HFD to Std diet added with ethanolic extract, to a value different from that of the group changed to Std diet (*P* < 0.05).

**Table 2:** Insulin tolerance test in hamsters fed HFD and experimental diets.

Groups and diets	% Glucose decrease/min^∗^
Std. diet (4 weeks)	4,58 ± 2,19ˆa,c
HFD (4 weeks)	0,65 ± 1,82ˆb
HFD + 3 mg% Ezetimibe	2,46 ± 0,41ˆa,d
HFD + 0.2% ethanolic extract	2,09 ± 0,96ˆa,e
HFD→Std. diet	2,55 ± 1,02ˆa,f
HDF→Std. diet + 3 mg% Ezetimibe	3,86 ± 0,76ˆc,d,e,f
HFD→Std. diet + 0.2% ethanolic extract	5,96 ± 0,66ˆc

*^∗^Mean ± SD, n = 4 per group. Different letter means significant difference (P < 0.05).*

### Liver Weight and Lipids

**FIGURE 4:**
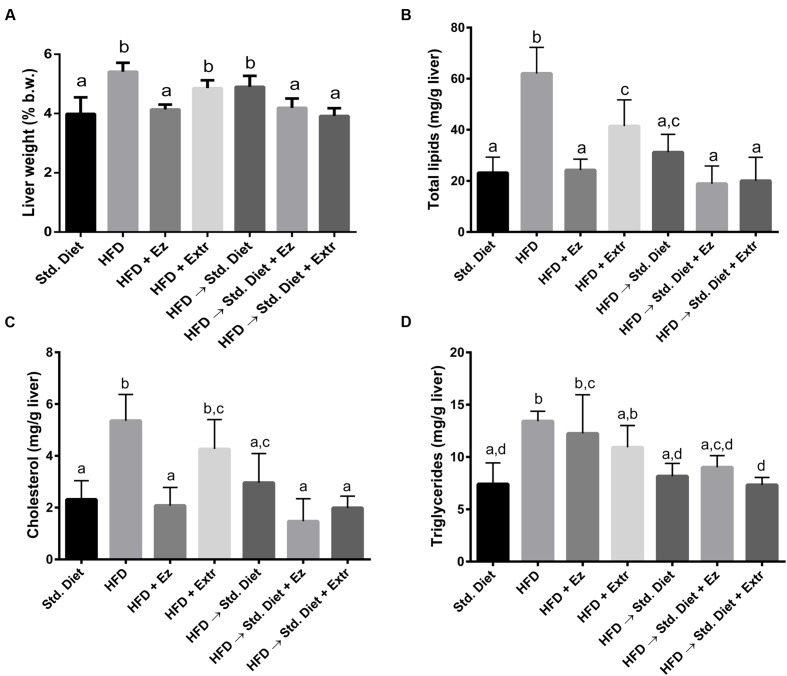
**Liver weight and liver lipids concentration in hamsters fed HFD for 2 weeks and Std diet or HFD diet with or without creosote bush extract or EZ for another 2 weeks. (A)** Liver weight, **(B)** total lipids, **(C)** cholesterol, and **(D)** triglycerides. Mean ± SD, *n* = 6 per group. Different letter between columns means significant difference (*P* < 0.05).

In relation to liver weight (**Figure 4A**), the group with HFD showed the highest values in absolute and percent liver weight, being different from the group with only Std diet (*P* < 0.05). The addition of EZ to HFD reduced liver weight to control values (*P* < 0.05). Liver weight from animals on HFD and creosote bush extract were lower, but did not significantly differ from the ones on HFD. The change from HFD to Std diet for 2 weeks did not significantly reduced liver weight. However, addition of EZ or extract returned liver weight to control values (Std diet group).

Total lipids (**Figure 4B**) were highest in the group on HFD. EZ decreased (*P* < 0.05) this parameter to control levels, whereas extract, although significantly reduced total lipids, did not reach control values. The change from HFD to Std diet also decrease total lipids to levels that did not significantly differ from control levels. Addition of EZ or extract to Std diet, although showed lower total lipids, were not significantly different from only Std diet. With cholesterol (**Figure 4C**) similar changes occurred, i.e., HFD induced higher concentration that were lowered by EZ, but the extract did not reach a significant difference with the HFD group. The change to Std diet without additions significantly reduced liver cholesterol concentrations, but none of the two additions made a significant difference from control. Hepatic TGs (**Figure 4D**) were higher with HFD and with HFD and EZ, respect to Std diet. Extract addition induced TG levels that also were not different from those with only HFD, however, values were neither significantly different from those in the group with only Std diet. The change to Std diet with or without addition returned TG levels to control (Std diet) concentrations.

### Liver Peroxidation and Antioxidant Capacity

**FIGURE 5:**
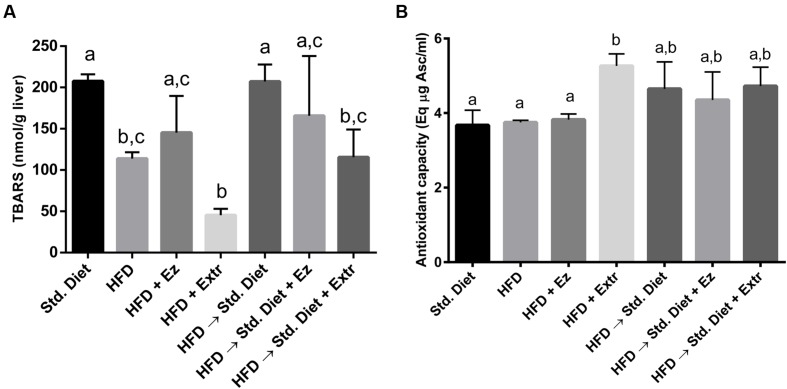
**Liver peroxidation (A) and antioxidant capacity (B) in hamsters fed HFD for 2 weeks and Std diet or HFD diet with or without creosote bush extract or EZ for another 2 weeks**. Mean ± SD, *n* = 6 per group. Different letter between columns means significant difference (*P* < 0.05).

As seen in **Figure 5**, unexpectedly, TBARS were twice as high in the control group than in the HFD one (*P* < 0.05). The group with HFD added with EZ, although with higher concentrations, did not statistically differ from the one on HFD. The addition of extract induced the lowest concentrations, although not significantly different from those in the HFD group. The change to Std diet induce again high TBARS to levels similar to Std diet for 4 weeks. Addition of EZ did not reduced TBARS, whereas extract addition significantly lowered this parameter.

Antioxidant capacity measured as delayed oxidation of DPPH, was not significantly different in control, HFD and HFD with EZ groups, only the one with addition of extract statistically (*P* < 0.05) increased the liver antioxidant capacity. The change to Std diet showed high values of antioxidant capacity that were not modified by EZ or extract additions, but neither were statistically different from control nor HFD values.

### Neutral Fecal Sterols

**FIGURE 6:**
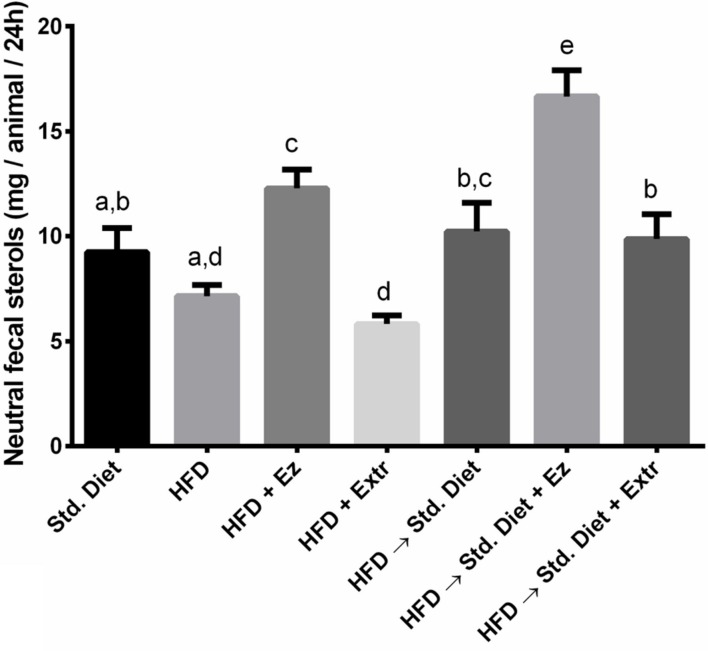
**Neutral fecal sterols in hamsters fed HFD for 2 weeks and Std diet or HFD diet with or without creosote bush extract or EZ for another 2 weeks**. Mean ± SD of three samples from pool of 48 h feces. Different letter between columns means significant difference (*P* < 0.05).

As shown in **Figure 6**, there was a significant (*P* < 0.05) increase in neutral fecal sterols in the groups with EZ, when compare with their own controls (HFD and HFD→Std diet groups). In the groups that received extract, fecal sterols were not significantly different from their own controls, which indicates that the extract effect is not related to a reduction of intestinal cholesterol absorption or increased excretion.

## Discussion

The Syrian golden hamster (*Mesocricetus auratus*) has a cholesterol and lipoprotein metabolism similar to that of humans (Bravo et al., 1994). This characteristic makes it an excellent model for studies on dyslipidemia and hypercholesterolemia, because changes in plasma lipids can be induced by specific dietary formulations, like the high fat and cholesterol diet (van Heek et al., 2001; Valasek et al., 2008; Zhang Z. et al., 2009). The present study also shows that a diet based on lard and cholesterol is effective in inducing some of the MS parameters, such as increased body weight, high plasma and hepatic lipids and low sensitivity to insulin. In general, the addition of creosote bush ethanolic extract to the HFD decreases plasma and hepatic lipids, increases the sensitivity to insulin and normalizes plasma insulin and leptin levels.

In humans, prevalence of MS is slightly higher in women than in men (Marquez-Sandoval et al., 2011; Beigh and Jain, 2012; Beltran-Sanchez et al., 2013). In the present study, only males were used, since the response to high fat diet is more pronounced in males than in females, with higher plasma total cholesterol and TG (Morise et al., 2006). In another study, hamsters of different sex and age were fed a high-calorie diet, dyslipidemia developed in males, irrespective of age, whereas in females only developed with aging (Zagayko et al., 2015). Nonetheless, it remains to be established whether the extract of *L. tridentata* has the same effects in females as in males.

Since the treatment of type 2 diabetes and MS in humans usually involves diet, exercise, and pharmacological therapy, in the present study the further effect of creosote bush extract on the change to a less caloric diet, such as maintenance diet, was analyzed. Several parameters, such as body weight, epididymal fat depots, plasma cholesterol, insulin, leptin, sensitivity to insulin, percent liver weight and peroxidation exhibited a further significant improvement with the change of diet and the addition of extract, in a 2 weeks treatment period, than with only a change to a less caloric diet, as the Std diet.

After 2 weeks of treatments, food consumption was similar among groups. The exception was the one fed Std diet with EZ, that exhibited the highest consumption of all, but despite that, it showed a body weight similar to that of the HFD animals. The epididymal fat pads, which reflect body adiposity, increased in hamsters fed HFD. EZ had no effect in this parameter, as previously published in hamsters (van Heek et al., 2001). The addition of creosote bush ethanolic extract to the HFD or to the Std Diet, induced the lowest body weight and adiposity of all groups, but not significantly different from the control group with Std diet for 4 weeks. The creosote bush ethanolic extract could have reduced nutrient absorption or accelerated the metabolism, since these groups gained less weight despite similar food consumption. In the short term this seems a beneficial effect, however, in the long term these could be a toxic one, also considering that excessive use of creosote bush can lead to hepatic and renal toxicity (Gordon et al., 1995; Sheikh et al., 1997).

Both EZ and creosote bush extract reduce plasma TGs and cholesterol, when added to the HFD. This shows a beneficial effect of the extract on components of the MS. A similar result on TGs was reported to be induced by Masoprocol (NDGA) in streptozotocin treated diabetic rats (Reed et al., 1999). EZ has previously been reported to reduce both parameters in hamsters on a high fat and cholesterol diet (van Heek et al., 2001; Valasek et al., 2008). HDL cholesterol was reduced by EZ in the HFD, as reported by van Heek et al. (2001), but not by the extract. The change to Std diet with or without additions, also decreases cholesterol, particularly with extract addition. In relation to TGs, the change to Std diet had no effect, and EZ or extract did not reduce this parameter significantly. The differential effect of extract and EZ when added to HFD and Std diet after HFD, may be due to differences in the type of lipids between diets, rather than in the quantity, since the fat content of the Std diet was 3.4%, according to a proximal chemical analysis. Whatever the reason, the Std diet used induced a high TG concentration.

Other characteristics of the MS are hyperinsulinemia, hyperleptinemia, and insulin resistance. These three parameters were present in the hamsters fed the HFD. The main physiological cause for leptin hypersecretion is diet-induced expansion of adipocytes. Circulating leptin levels are directly proportional to the amount of body energy stores. As energy storage capacity in adipocytes is exceeded during the period between the onset of overweight/obesity and the start of the MS, leptin resistance develops (Moon et al., 2013). This lack of action leads to overfeeding and generalized steatosis and lipotoxicity that leads to insulin resistance (Unger et al., 2010). The creosote bush ethanolic extract added to the HFD decreased serum leptin and insulin to normal values and insulin sensitivity to levels not different from animals with Std diet. The change from HFD to Std diet did not affect serum insulin, but leptin was reduced. The addition of extract to Std diet decreases both hormones to normal levels, as when added to the HFD. These effects could be related to lower weight gain and lower adiposity, as shown by low epididymal fat pads in both groups with extract, but not due to lower food consumption. Several phenolic compounds, such as resveratrol, oleuropein, and myricetin, as well as some precursors or derivatives of phenols, such as polydatin, have been found to reduce the level of circulating leptin in a large range of *in vivo* studies, using different types of models (Aragonés et al., 2016). In addition to phenolic compounds, other plant secondary metabolites, such as isothiocyanates and some terpenoids including thymol, saponins, and lycopenes among others, have also been shown to be effective in reducing leptin levels when administered to rodents (Aragonés et al., 2016). The mechanisms of action of these compounds have not been established for the majority of them. Resveratrol increases AMPK activity, reducing lipogenesis and increasing lipid oxidation (Zhang B.B. et al., 2009). Others, like some coumarins, flavonoids and polyphenols from fruit juices, could act as PPAR agonists or even improve leptin transport through the brain blood barrier (Aragonés et al., 2016), enhancing the oxidation of surplus fatty acids and reducing lipotoxicity (Unger et al., 2010). It remains to be established how creosote bush extract, probably through NDGA, reduces leptin resistance and improves insulin sensitivity. EZ had no significant effect in leptin and insulin, as reported by van Heek et al. (2001), although insulin sensitivity did improve.

The HFD increased liver size and lipids as previously published (van Heek et al., 2001; Valasek et al., 2008). In our study, liver weight and lipids did not significantly decrease in hamsters fed HFD added with creosote bush ethanolic extract, except for total lipids. However, lower values were observed in TGs and cholesterol, with hepatic TGs concentration not different from the level in animals on Std diet. Zhang et al. (2015) reported a decrease in hepatic TGs with NDGA, the main metabolite of creosote bush, in rats with a high fructose diet. EZ at the level used was more effective in decreasing liver weight, total lipids and cholesterol, a result that agrees with previous reports in hamster (van Heek et al., 2001; Valasek et al., 2008). The change from HFD to Std diet, with or without added EZ or extract, was enough to return liver parameters to control values, although the lowest values were those with the addition of extract or EZ.

Oxidative stress has been implicated in the development of MS and type 2 diabetes (Hopps et al., 2010). Since the main metabolite of the extract is NDGA, a recognized antioxidant, peroxidation and antioxidant capacity in the liver were analyzed. Liver peroxidation, estimated as TBARS, was higher with the Std diet than with the HFD, while antioxidant capacity was similar with both diets. This TBARS reduction with the HFD may be due to differences in type of lipids between diets. In mice fed a diet with 8% fish oil, which contains highly unsaturated fatty acids, a significantly higher hepatic concentrations of TBARS was found than with 10% lard (Ibrahim et al., 1997). This indicates that in the HFD fed hamster, oxidative stress, at least in the liver, is not associated with signs of the MS.

As expected, creosote bush ethanolic extract decreased peroxidation with HFD and with the change to Std diet, and increased antioxidant capacity with HFD. EZ had no effect on these parameters, and it is known that its mechanism of action is through inhibiting cholesterol absorption (van Heek et al., 2001; Valasek et al., 2008). According to this, neutral fecal sterols were increased by EZ in the present work. Creosote bush ethanolic extract has been reported to have antibiotic actions (Mabry et al., 1979; Argueta, 1994), which could have modified intestinal microbiota. However, it does not affect cholesterol absorption as indicated by neutral fecal sterols.

At least one active principle of creosote bush ethanolic extract must be NDGA, which in the present study was found to constitute 8.9% of our ethanolic extract. Belmares et al. (1981) have reported that NDGA was 26% of ethanol extract of leaves and twigs. The difference with our analysis may be due to the methodology used. Belmares group employed gas chromatography of trimethylsilyl derivatives, so that besides NDGA, also several *O*-methylated derivatives, such as guaiaretic acid, present in the extract were accounted for as NDGA. Components of the extract include other lignans and flavonoids (Timmermann, 1981; Vázquez-Yanes et al., 1999; Gnabre et al., 2015). During many years, the beneficial effects of flavonoids on health were ascribed to their antioxidant capacity. They can also improve dyslipidemia by modulating lipid absorption and lipogenesis (Galleano et al., 2012). It remains to be established whether the “agua de uso” from creosote bush traditionally used in diabetes treatment exerts the same effects as the ethanolic extract. Our results and those of Zhang et al. (2015) with fructose fed rats and NDGA, suggest that creosote bush derivatives also have potential utility in the treatment of MS.

## Conclusion

The beneficial effects of creosote bush ethanolic extract in the HFD hamster model that develops some signs of the MS are to reduce plasma TGs, total cholesterol, insulin and leptin, and improve insulin sensitivity. These effects are associated with a lower lipid peroxidation and an increase in antioxidant capacity in the liver. When the HFD diet is changed to Std diet, plasma glucose and cholesterol, serum leptin, insulin sensitivity and liver lipids improve. The addition of creosote bush ethanolic extract to Std diet, further reduces body and epididymal fat pads weight, plasma glucose and cholesterol, serum insulin and leptin, liver weight and peroxidation, also increasing sensitivity to insulin. This suggests that the ethanolic extract of *L. tridentata* could be useful in the treatment of the MS.

## Author Contributions

GD and RC-V performed experiments, analyzed the data and wrote the manuscript. MR-C performed experiments and amended the paper. AA-C amended the paper.

## Conflict of Interest Statement

The authors declare that the research was conducted in the absence of any commercial or financial relationships that could be construed as a potential conflict of interest.

## ABBREVIATIONS

HFD: high fat diet
IDF: International Diabetes Federation
MS: metabolic syndrome
NDGA: nordihydroguaiaretic acid
TG: triglycerides.
